# Association between viral suppression during the third trimester of pregnancy and unintended pregnancy among women on antiretroviral therapy: Results from the 2019 antenatal HIV Sentinel Survey, South Africa

**DOI:** 10.1371/journal.pone.0265124

**Published:** 2022-03-17

**Authors:** Selamawit Woldesenbet, Tendesayi Kufa, Samuel Manda, Kassahun Ayalew, Carl Lombard, Mireille Cheyip, Adrian Puren

**Affiliations:** 1 Center for HIV and STI, National Institute for Communicable Diseases, Johannesburg, South Africa; 2 School of Public Health, University of the Witwatersrand, Johannesburg, South Africa; 3 Biostatistics Unit, South African Medical Research Council, Pretoria, South Africa; 4 Department of Statistics, University of Pretoria, Pretoria, South Africa; 5 Biostatistics Unit, South African Medical Research Council, Cape Town, South Africa; 6 Strategic Information Unit, Centers for Disease Control and Prevention, Pretoria, South Africa; 7 Division of Virology, School of Pathology, University of the Witwatersrand, Johannesburg, South Africa; University of Cape Town, SOUTH AFRICA

## Abstract

**Objectives:**

About half of the pregnancies among women living with HIV (WLWH) receiving antiretroviral therapy (ART) in sub-Saharan African countries are reported to be unintended. Unintended pregnancy is associated with late initiation of antenatal care (ANC), and may delay provision of viral load monitoring services, antenatal adherence counselling and support, and other services that promote sustained viral suppression throughout pregnancy. This study examines the association between unsuppressed viral load during the third trimester of pregnancy and unintended pregnancy among women who initiated ART before pregnancy.

**Methods:**

This was an analysis of data from a national antenatal survey conducted at 1 589 public health facilities in South Africa between 1 October and 15 November 2019. Consenting pregnant women aged 15–49 years attending ANC during the survey period were enrolled. Demographic and clinical data were collected through interview and medical record review. Pregnancy intention was assessed using two questions from the London Measure of Unplanned Pregnancy, and responses were categorized as “unintended,” “undecided,” and “intended.” Blood specimens were collected from all women and tested for HIV; and if positive, a viral load test was performed. A survey domain-based poisson regression model examined the association between unsuppressed viral load during the third trimester of pregnancy and unintended pregnancy among women who initiated ART before pregnancy. Viral suppression was defined as viral load <50 copies/mL.

**Results:**

Of 10 901 WLWH with viral load data available, 63.3% (95% confidence interval (CI): 62.4%-64.1%) were virally suppressed. Among the 2 681 women (representing 24.1% of all WLWH with viral load data) who initiated ART before pregnancy and were in their third trimester at the time of enrolment, 74.4% (95% CI: 73.0%-75.8%) were virally suppressed. In the same population, the proportion virally suppressed was lower among women whose current pregnancies were unintended (72.1%, 95% CI: 70.1%-74.1%) compared to women whose pregnancies were intended (78.3%, 95% CI: 75.9%-80.5%). In multivariable analyses adjusted for age, gravity, marital status, education, location of facility and syphilis status, unintended pregnancy was associated with unsuppressed viral load during the third trimester (adjusted relative risk: 1.3, 95% CI: 1.1–1.4) among women who initiated ART before pregnancy.

**Conclusion:**

The identified association between unsuppressed viral load and unintended pregnancy among pregnant women who initiated ART before pregnancy highlights the need to strengthen routine assessment of fertility preferences and provision of contraceptive services to reproductive age WLWH receiving ART.

## Introduction

The Joint United Nations Programme on HIV/AIDS (UNAIDS) estimated that in 2020, 150 000 children globally acquired HIV from their mothers [1]. Without intervention, 20–35% of children born to pregnant women living with HIV become infected at birth [2]. Early initiation of antiretroviral therapy (ART) improves viral suppression at delivery and reduces the risk of vertical HIV transmission [3]. Pre-pregnancy ART initiation, and viral suppression (viral load <50 copies/ml) throughout pregnancy and breastfeeding is the most effective intervention to eliminate the risk of vertical HIV transmission [3].

Globally, although ART initiation before pregnancy has substantially improved, a large percentage (up to 30%) of women receiving ART conceive with unsuppressed viral load [4–9]. Factors such as unintended pregnancy, low uptake of contraceptives, and poor access to safer conception services may contribute to the high prevalence of pregnancy among women with unsuppressed viral load. The South African guideline for safer conception recommends all couples who intend to become pregnant should test for HIV before pregnancy and if positive should attain viral suppression before trying to achieve pregnancy [10]. Planning pregnancy offers those who are unaware of their HIV status the opportunity to test for HIV, and initiate ART (if HIV-positive) before pregnancy. Conversely, unintended pregnancy is associated with late antenatal care (ANC) booking, delayed initiation of ART during pregnancy, and increased levels of stress [11–13]. Studies have shown increased level of stress associated with unintended pregnancy can be a threat to the physical and mental health of the women and the well-being of the unborn child [14, 15]. For women who initiate ART before pregnancy, late initiation of ANC associated with unintended pregnancy may delay provision of viral load monitoring services, antenatal adherence counselling and support, and other services that promote sustained viral suppression throughout pregnancy [16].

South Africa has conducted Antenatal HIV Sentinel Surveys since 1990. The surveys conducted prior to 2017 primarily monitored HIV prevalence. Since 2017, the survey tracks the progress towards meeting the UNAIDS 90-90-90 and 95-95-95 targets, which aim to ensure 90% and 95% of people living with HIV know their HIV status, 90% and 95% of people diagnosed with HIV receive sustained ART and 90% and 95% of people on ART have viral suppression by 2020 and 2030, respectively. This translates to an overall target of 73% and 86% viral suppression among all people living with HIV in 2020 and 2030, respectively. In the 2017 Antenatal HIV Sentinel Survey, it was found that only 56.2% of pregnant women had viral suppression (viral load ≤50 copies/ml), and late ANC booking and late initiation of ART were associated with poor viral suppression [17]. This highlighted the need to closely monitor viral load, strengthen counselling, and support services for ART adherence. An important public health concern is the high level of unintended pregnancy among women living with HIV (WLWH) and its potential role in delaying viral suppression during pregnancy [18].

Studies that have been done to assess the magnitude and relationship between pregnancy intentions and pregnancy outcomes have shown the high prevalence of unintended pregnancy in sub-Saharan African (SSA) countries and the strong correlation between unintended pregnancy and late ANC booking [11–16, 18, 19]. A handful of studies have also shown correlation between elevated viral load during pregnancy and unintended pregnancy [16, 19, 20]. However, these studies either were done in developed countries or only assessed the relationship between viral load level at entry to ANC and unintended pregnancy. Several studies have shown vertical HIV transmission is more likely to occur during the last trimester of pregnancy than in the early stages of pregnancy [21–26]. Given the greater likelihood of vertical HIV transmission during late pregnancy, better understanding of the relationship between viral load level in the last trimester of pregnancy and unintended pregnancy is crucial for public health practice, policies and strategies that target improvement in viral suppression among pregnant women.

Using data from the 2019 Antenatal HIV Sentinel Survey, this study describes viral suppression among pregnant women living with HIV and examines the association between unsuppressed viral load during the third trimester of pregnancy and unintended pregnancy among women who initiated ART before pregnancy. At the time of this study, the first line ART regimen for people living with HIV was an Efavirenz-based regimen.

## Methods

### Study design and participants

This study used data from the national Antenatal HIV Sentinel Survey conducted among pregnant women between the ages of 15–49 years attending ANC in public health facilities in South Africa between 1 October and 15 November 2019. The antenatal survey is a cross-sectional survey conducted in all 52 districts of South Africa every 1–2 years. The 2019 survey enrolled 41 598 pregnant women attending either first ANC visit or follow-up visit in the current pregnancy—from 1 589 sentinel sites.

For the current study, only WLWH with viral load data were included: specifically, in the first part of the analysis describing viral suppression, all WLWH with viral load data were included. In the second part of the analysis assessing association between viral suppression during the third trimester and unintended pregnancy, women who initiated ART before pregnancy and were in their third trimester at the time of enrolment in the survey were included. The sample size collected was adequate to estimate viral suppression among all WLWH at national level within 1–2% precision. Assumptions for this calculation included expected viral suppression rate of 55–60% (using viral suppression cut off point of <50 copies/mL), HIV prevalence of 30%, design effect of 1.5, using 95% confidence interval (CI), and 10% error rate. The sample size also allowed the detection of a ≥6% difference in viral suppression between participants who reported their pregnancy was intended and those who reported their pregnancy was unintended, among women initiating ART before pregnancy. The assumptions for this calculation included: power 80%, significance level of 0.05, proportion of exposed versus unexposed equal to 1, design effect of 1.5, 65% of WLWH initiate ART before pregnancy and viral suppression of 75% at the third trimester among women with intended pregnancy. The sample size was not adequate to assess association between unintended pregnancy and viral suppression among women initiating ART during pregnancy.

### Sampling and data collection procedures

Sentinel sites were selected from each of the 52 districts in South Africa using stratified (by district) probability proportional to size (PPS) cluster sampling method using the ANC volume of sentinel sites as proxy for size. During the study period, consenting pregnant women aged 15–49 years, attending the antenatal clinic for the first time or for follow-up visits during their current pregnancy were consecutively enrolled (regardless of HIV or ART status) until either the required sample size was reached or until the end of the study. Health workers providing ANC services in the selected facilities collected socio-demographic data (including participant’s education level, race, relationship with the father of the child, gravidity, and pregnancy intent) through interview. Data on age of the woman, gestational age on the day of interview, gestational age at first ANC visit, HIV test results, syphilis test results, timing of HIV diagnosis, and ART initiation were extracted from participants’ medical records. Pregnancy intent was assessed using two questions adopted from the London Measure of Unplanned Pregnancy (LMUP)–a five item (Likert type scale) validated measure of the degree of intention of pregnancy (S1 Table) [27]. The two items included in this study were LMUP item 3 “Just before I became pregnant…” with response options “I intended to get pregnant,” “My intentions kept changing,” and “I did not intend to get pregnant” and LMUP item 5 “Before I became pregnant…” with response options “The father of the child and I had agreed that we would like me to be pregnant,” “The father of the child and I had discussed having children together, but hadn’t agreed for me to get pregnant,” and “We never discussed having children together.” A blood specimen was taken from each woman regardless of prior knowledge of HIV status or ART initiation. Detailed description of the study procedures is presented in the main survey report [28].

### HIV viral load testing

Blood specimens were tested for HIV using serial immunoassay (IA) tests: screening assay (IA-1) and confirmatory assay (IA-2). Specimens that were reactive on both IA-1 and IA-2 were classified as HIV-positive and tested for viral load. A detailed description of the HIV serological testing procedures is provided in the main report [28].

HIV viral load testing on all confirmed HIV-positive (plasma) specimens was carried out using the COBAS AmpliPrep/COBAS TaqMan (CAP/CTM) HIV-1 Quantitative test (Roche Molecular Systems, Inc., Branchburg, New Jersey, USA) following the manufacturer’s instructions. In addition to the internal control, the assay included external controls in each run, a low positive control, a high positive control, and a negative control. The viral load tests were done on specimens collected at enrolment in the survey. Participants were at different gestational age at enrolment in the survey, therefore the viral load results reflect viral load of women who were at different gestational age.

### Description of variables and outcome measures

Two main outcome measures were assessed.

#### Overall viral suppression

For the part of the analysis aiming to describe viral suppression, the main outcome was viral suppression (defined as viral load <50 copies/ml) among all WLWH with viral load data.

#### Association between viral suppression and unintended pregnancy

This part of the analysis was restricted to women who initiated ART before pregnancy and were in their third trimester at the time of enrolment. The main outcome for this section of the analysis was viral suppression (defined as viral load <50 copies/ml) during the third trimester (gestational age 28–42 weeks). The main exposure variable was unintended pregnancy among women initiated on ART before pregnancy. The two pregnancy intent questions were classified into the following three categories: 1) unintended pregnancy by both questions, 2) intended pregnancy by both questions and 3) ‘undecided about pregnancy’ which included responses where one response indicated the pregnancy was intended and the other response did not (S1 Table). Other key exposure variables included syphilis status, the woman’s age, education, marital status, location of the health facility (i.e. urban/rural /peri-urban classification), and gravidity. Syphilis status was based on the latest Rapid Plasma Regain (RPR) test results extracted from the woman’s medical record. Syphilis test results were categorized into three categories: RPR positive, RPR negative, and other–the option ‘other’ included syphilis status not in file or not recorded and pending results. All women attending first ANC visit on the day of the survey, excluding those who attended facilities that provide point-of-care syphilis testing, would have pending syphilis test result, as specimens for syphilis tests are collected at first ANC visit and sent to the local laboratories for testing, and results are returned in the next visit.

### Data analysis

Data were analyzed using STATA 14 (StataCorp. 2015. Stata Statistical Software: Release 14. College Station, TX: StataCorp LP) [29]. Analysis took into account the survey design (i.e. clustering within facilities, and stratification by district) and was weighted for the Statistics South Africa mid-year population size of women of reproductive age (15–49 years) in 2019 at province level and for sample size realization. Survey domain analysis was used for all subpopulation level analysis. Given that sites were sampled using PPS, and that the sampling period was fixed, this provided a self-weighted sample at district level. A finite population correction factor was added to adjust for the >5% of facilities sampled without replacement from a finite population of about 4 000 public facilities.

#### Analysis describing overall viral suppression

For the first part of the analysis describing the overall viral suppression among pregnant women living with HIV, all WLWH (per IA test) who consented to participate in the survey, and had completed questionnaire and valid viral load test results were included. The median viral load and interquartile range (IQR), and overall proportion with viral suppression was reported at national level and among women that initiated ART before pregnancy. In addition, viral suppression was reported in stratified groups by gestational age (i.e. as first trimester: ≤12 weeks, second trimester: 13–27 weeks, and third trimester: 28–42 weeks) in the overall sample (i.e. among WLWH) as well as among women initiated on ART before pregnancy. All point estimates were reported with 95% confidence intervals (CIs).

#### Analysis assessing association between viral suppression and unintended pregnancy

For this part of the analysis, WLWH who were in their third trimester during the survey and who had initiated ART before pregnancy were included. Viral suppression and early (first trimester) ANC attendance was compared among participants whose pregnancy was intended, unintended, and undecided. Chi-square tests were used to test significant associations in stratified analysis. A survey domain based multivariable poisson regression model was fitted to examine the association between unsuppressed viral load during the third trimester and pregnancy intention. The robust poisson regression model was a preferred method for this study over the log-binomial regression or logistic regression model as poisson model is less sensitive to model misspecification and is appropriate for common outcomes [30]. The association between viral suppression and unintended pregnancy was assessed only during the third trimester of pregnancy because viral load during the third trimester is a good proxy for viral load at delivery, which is an important predictor of vertical HIV transmission [24, 25]. The main variable of interest (i.e. pregnancy intent: unintended pregnancy vs intended vs undecided) and other covariates significant at P value cut off point of 0.05 and variables that have ≥10% effect on the relative risk of the variable of interest were kept in the model. Factors considered to be mediators (such as timing of ANC booking) between unintended pregnancy and unsuppressed viral load, were not included in the model.

The multivariable analysis was restricted to those who initiated ART before pregnancy, because the mechanism by which unintended pregnancy affects viral suppression was thought to be different by timing of ART initiation. Among women initiating ART during pregnancy, unintended pregnancy is likely to be associated with delayed initiation of ART [19], whereas for women who have already initiated ART before pregnancy, unintended pregnancy does not affect timing of ART initiation. Thus, the multivariable analysis assessing association between viral suppression and unintended pregnancy was fitted separately for women initiating ART before pregnancy. Analysis examining association between viral suppression and pregnancy intention was not done for women initiating ART during pregnancy due to inadequate sample size.

### Ethical considerations

Participation in the survey was voluntary, requiring written informed consent. A waiver of parental permission has been obtained from the local IRB for participants in the age between 15 and 17 years [31]. To protect the confidentiality of participants’ information, the data collection form was submitted without patient identification. Participants could withdraw from the study at any time and this did not influence their treatment. Participants were not compensated for their participation. Ethical approval was obtained from the University of the Witwatersrand Human Research Ethics Committee (Medical), and the nine provincial health research ethics committees. The study was reviewed in accordance with the U.S. Centers for Disease Control and Prevention (CDC) human research protection procedures and determined to be research, but CDC investigators did not interact with human subjects or have access to identifiable data or specimens for research purposes.

## Results

### The demographic and clinical characteristics of participants

**Fig 1:**
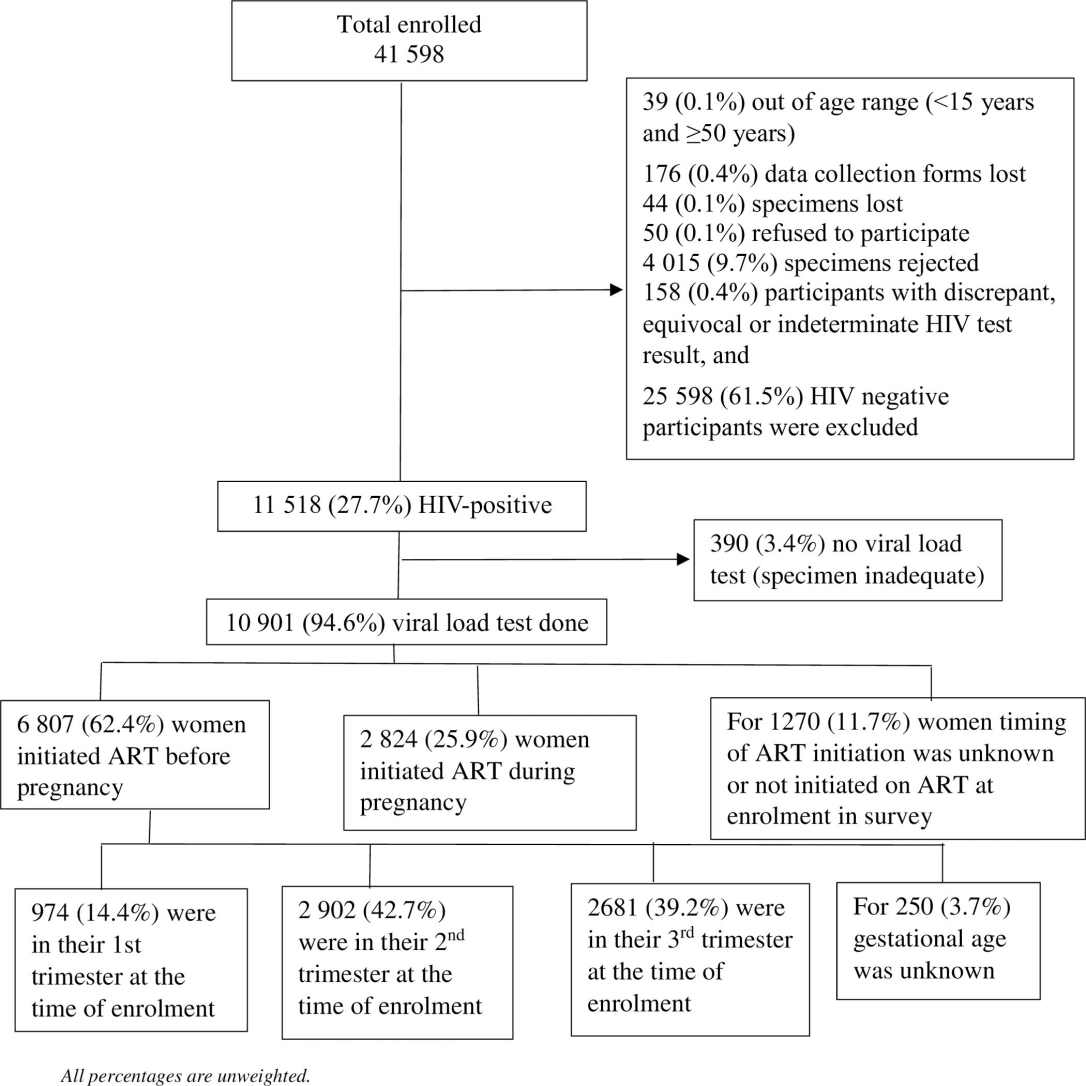
Flow chart of observations excluded from the analysis, the 2019 antenatal HIV Sentinel Survey, South Africa.

Of a total of 41 598 participants enrolled in the survey, 11 518 participants were HIV-positive (by IA test performed for the survey), of which 94.6% (10 901) participants had a specimen tested for viral load, and were therefore included in the analysis describing viral suppression (Fig 1). Of the 10 901 women with viral load data available, 62.4% (6 807) initiated ART before pregnancy, of whom 39.2% (2681) (representing 24.1% of all WLWH with viral load data) were in their third trimester at the time of enrolment therefore were included in the analysis assessing association between viral suppression during the third trimester and unintended pregnancy among women initiated ART before pregnancy. There was no statistically significant difference in demographic characteristics (such as age, marital status, location of facility, and gestational age) and the prevalence of unintended pregnancy between participants with missing viral load data and those with viral load data.

The demographic and clinical characteristics of participants are presented in Table 1. Most characteristics were similar between all WLWH with viral load data and participants who initiated ART before pregnancy and were in their third trimester at enrolment. The characteristics described in this section were observed in both groups. The majority (>75%) of participants were between 25 and 49 years old and had been pregnant at least once before the current pregnancy. More than 95% of participants were Black. About one-half of participants reported their pregnancy was unintended. More than 3% of participants tested positive for syphilis during pregnancy. Almost all syphilis positive participants (>95%) received syphilis treatment as part of routine care during a prior or current ANC visit.

**Table 1:** Demographic and clinical characteristics of participants, antenatal HIV Sentinel Survey, 2019, South Africa.

Description	All participants with viral load data (n = 10 901)* Number (%)	Participants initiated on ART before pregnancy and were in their third trimester at the time of enrolment (n = 2681)* Number (%)
Age in years		
**15–19**	447 (4.2)	63 (2.4)
**20–24**	1 823 (18.0)	352 (13.5)
**25–29**	2 974 (29.4)	693 (27.7)
**30–34**	2 773 (27.9)	811 (32.8)
**35–49**	2 027 (20.5)	587 (23.7)
**Population group**		
**Black African**	10 398 (96.5)	2581 (97.6)
**Other (Coloured, White, Asian)**	445 (3.5)	88 (3.4)
**Education**		
**No or primary**	1 473 (14.4)	349 (13.2)
**Secondary**	8 215 (75.7)	2082 (77.4)
**Tertiary**	1 050 (10.0)	238 (9.4)
**Marital status**		
**Married**	1 548 (14.4)	388 (14.6)
**Cohabiting**	3 146 (31.2)	774 (30.6)
**In a non-cohabiting relationship**	5 661 (50.9)	1389 (50.9)
**Single**	375 (3.5)	106 (3.9)
**Province**		
**Eastern Cape**	1 967 (12.2)	440 (11.2)
**Free State**	918 (5.5)	259 (6.4)
**Gauteng**	1 504 (25.2)	314 (21.8)
**KwaZulu-Natal**	3 306 (28.7)	924 (33.4)
**Limpopo**	509 (6.3)	119 (6.1)
**Mpumalanga**	953 (7.8)	247 (8.2)
**North West**	710 (6.0)	162 (5.7)
**Northern Cape**	336 (1.5)	78 (1.4)
**Western Cape**	698 (7.0)	138 (5.8)
**Location of facility**		
**Urban**	6 446 (63.1)	1467 (58.4)
**Rural**	3 488 (28.8)	941 (32.3)
**Peri-urban**	967 (8.1)	273 (9.3)
**Gravidity**		
**Primigravida (1)**	1 666 (15.3)	252 (9.3)
**Multigravida (2+)**	8 996 (84.7)	2392 (90.7)
**Pregnancy intention**		
**Intended**	3 351 (34.0)	818 (33.2)
**Undecided**	1 456 (14.2)	354 (13.9)
**Unintended**	5 382 (51.8)	1379 (52.9)
**Syphilis infection status during pregnancy**		
**Positive**	378 (3.6)	86 (3.2)
**Negative**	8 164 (80.2)	2393 (91.7)
**Other (result pending, not in file)**	1 459 (16.2)	117 (5.1)
**Median gestational age at first ANC† visit (IQR)††**	15 weeks (IQR: 10–20)	17 weeks (IQR: 12–22)

*This table presents the characteristics of women with viral load data (n = 10 901) and women who initiated ART before pregnancy with viral load data during the third trimester (n = 2681). The characteristics of both groups of participants needed to be described in this table as each were the study target population for the different sections of the analysis. As one data is a subset of the other, the focus is not to compare these two groups but to describe each data independently. Weighted percentages; missing data not included when calculating percentages.

† ANC: antenatal care.

†† IQR: interquartile range.

### Viral suppression among all women with viral load data

In the overall sample (n = 10 901), close to half (45.1%) of WLWH had undetectable (i.e. below assay limit of quantification) viral load. Among the remaining 54.9% (n = 5984) of women with detectable viral load, the median viral load was 235 copies/mL (IQR: 66–3 819). Viral suppression was achieved by 63.3% (95% CI: 62.4%-64.1%) of participants including those with undetectable viral load (note: participants with undetectable viral load had below 50 copies /mL therefore were counted with groups that have achieved viral suppression) (Table 2). Of the participants who had viral load data, 14.1%, 44.4%, and 41.5% were in their first, second and third trimester, respectively, at the time of enrolment. Viral suppression was 57.7% (95% CI: 55.8%-59.6%) among participants who were in their first trimester, 61.6% (95% CI: 60.3%-62.9%) among participants who were in their second trimester, and 67.4% (95% CI: 66.1%-68.6%) among participants who were in their third trimester.

**Table 2:** Viral suppression of women by gestational age and among women initiated on ART before pregnancy in the 2019 South African antenatal HIV Sentinel Survey.

Viral suppression <50 cps/mL (95% CI)*		
Description	All women with viral load data % (95% CI) **	Among women initiated on ART before pregnancy % (95% CI)
Overall	63.3 (62.4–64.1)	73.3 (72.3–74.2)
Among participants in their first trimester	57.7 (55.8–59.6)	73.3 (71.1–75.4)
Among participants in their second trimester	61.6 (60.3–62.9)	72.5 (71.0–73.9)
Among participants in their third trimester	67.4 (66.1–68.6)	74.4 (73.0–75.8)

*The denominators for the first column from top row to bottom row are: 10 901, 1460, 4604 and 4327 respectively. The denominators for the second column from top row to bottom row are: 6807, 974, 2902, and 2681 respectively. Viral suppression among women initiated ART during pregnancy was not reported in this study as this topic is outside the scope of the current study, but as reported previously in the 2017 survey, in the 2019 survey as well, viral suppression was substantially lower (46.2%, 95% CI: 44.8%-47.7%) among women who initiated ART during pregnancy compared to those initiated ART before pregnancy (73.3%).

**CI: confidence interval.

### Viral suppression among women initiated on ART before pregnancy

Among women initiated on ART before pregnancy, viral suppression was 73.3% (95% CI: 72.3%-74.2%) regardless of gestational age at enrolment. In this population, viral suppression did not vary by gestational age: viral suppression was 73.3% (95% CI: 71.1%-75.4%) among women who were in their first trimester, 72.5% (95% CI: 71.0%-73.9%) and 74.4% (95% CI: 73.0%-75.8%) among women in their second and third trimester respectively (Table 2).

### Association between unsuppressed viral load in the third trimester and unintended pregnancy among women initiated on ART before pregnancy

Women whose pregnancy was unintended had significantly lower viral suppression during the third trimester (72.1%, 95% CI: 70.1%-74.1%) compared to women whose pregnancy was intended (78.3%, 95% CI: 75.9%-80.5%) (Table 3). Attendance of ANC in the first trimester was higher (30.1%) among women whose pregnancy was intended compared to women whose pregnancy was unintended (23.2%); and a higher percentage (13.1%) of participants whose pregnancy was unintended attended their first ANC visit in the third trimester compared to participants whose pregnancy was intended (7.8%) (p value <0.01).

**Table 3:** Association between unsuppressed viral load during the third trimester of pregnancy and unintended pregnancy among women who initiated ART before pregnancy in the 2019 antenatal survey.

	Sample distribution n = 2681^†^	Unadjusted relative risk (RR) (95% CI)^┼^	Adjusted RR (95% CI)
Pregnancy intention*			
Intended	818 (33.2)	Ref	Ref
Undecided	354 (13.9)	1.1 (0.9–1.3)	1.0 (0.9–1.3)
Unintended	1 379 (52.9)	1.3 (1.1–1.5)	1.3 (1.1–1.4)
**Age group (in years)**			
15–19	63 (2.2)	1.4 (1.0–1.8)	1.2 (0.9–1.7)
20–24	352 (12.5)	1.2 (1.0–1.5)	1.2 (1.0–1.4)
25–29	693 (25.7)	1.0 (0.9–1.2)	1.0 (0.9–1.2)
30–34	811 (30.4)	1.0 (0.8–1.1)	0.9 (0.8–1.1)
35–49	587 (22)	Ref	Ref
Age not reported	175 (7.2)	0.9 (0.7–1.1)	0.9 (0.7–1.2)
**Education**			
No or primary	349 (13.2)	1.5 (1.2–1.9)	1.5 (1.2–1.9)
Secondary	2 082 (77.4)	1.3 (1.0–1.5)	1.2 (1.0–1.5)
Tertiary	238 (9.4)	Ref	Ref
**Marital status**			
Married	388 (14.6)	Ref	Ref
Co-habiting	774 (30.7)	1.4 (1.2–1.7)	1.3 (1.1–1.6)
In a non-cohabiting relationship	1 389 (50.9)	1.3 (1.1–1.6)	1.2 (1.0–1.4)
Single	106 (3.9)	1.4 (1.0–1.8)	1.2 (0.9–1.7)
**Gravidity**			
Primigravida (1)	252 (9.3)	1.1 (0.9–1.3)	1.1 (0.9–1.3)
Multigravida (2+)	2 392 (90.7)	Ref	Ref
**Syphilis status**			
Negative	2 393 (91.7)	Ref	Ref
Positive	86 (3.7)	1.1 (0.8–1.5)	1.0 (0.8–1.4)
Result pending, not in file)	117 (5.1)	1.5 (1.2–1.9)	1.5 (1.2–1.8)
**Location of facility**			
Urban	1 467 (58.4)	Ref	Ref
Rural	941 (32.3)	1.3 (1.12–1.4)	1.3 (1.2–1.5)
Peri-urban	273 (9.3)	1.0 (0.9–1.3)	1.1 (0.9–1.3)

†90.2% (2 417/2 681) of observations included in multivariable analysis.

*as pregnancy intention has three categories, a test was conducted to assess the overall effect of pregnancy intention and the test showed the overall effect of ‘pregnancy intention’ was significant (P value <0.01).

┼CI: confidence interval.

In a bi-variable analysis, unintended pregnancy was associated with a 30% increase in the risk of unsuppressed viral load during the third trimester (Table 3). In a multivariable analysis (adjusting for age, education, marital status, gravidity, location of facility, and syphilis infection status), unintended pregnancy was significantly associated with unsuppressed viral load during the third trimester (adjusted relative risk—ARR: 1.3, 95% CI: 1.1–1.4). There was no significant association between being undecided about their pregnancy and viral suppression. Other covariates influential on unsuppressed viral load in multivariable analyses included: attending ANC in health facilities located in rural areas (compared to urban areas), no or primary education compared to tertiary education, being in a cohabiting relationship compared to being married, and pending syphilis result and results not in file compared to syphilis negative result–about 50% of pending syphilis results were for women who booked ANC on the day of the survey (i.e. during the third trimester).

## Discussion

This study aimed to determine viral suppression among pregnant women nationally and explore an association between viral suppression during the third trimester of pregnancy and unintended pregnancy among pregnant women who initiated ART before pregnancy using a national survey conducted in 2019 in South Africa. The findings showed that just under two-thirds of pregnant women living with HIV were virally suppressed, with higher viral suppression rates achieved among women who initiated ART before pregnancy. Unintended pregnancy was associated with an increase in the risk of unsuppressed viral load during the third trimester after controlling for demographic and clinical factors.

In this study, both overall viral suppression (63.3%) and viral suppression during the third trimester (67.4%) were below the UNAIDS target of 73% viral suppression in the overall sample. The UNAIDS target was achieved among women who initiated ART before pregnancy (74%) highlighting the importance of ART initiation before pregnancy. Viral suppression is the most important predictor of vertical HIV transmission. In high HIV burden countries like South Africa, viral suppression rates need to be higher than 90% in order to achieve the elimination of vertical HIV transmission target of reducing vertical HIV transmission case rates to 50/100 000 livebirths. According to Statistics South Africa and UNAIDS, in the year this survey was conducted (i.e. in 2019), 954 532 livebirths and 10 000 new child infections (vertical HIV transmission) occurred in South Africa which translates to about a 1000 vertical HIV transmission cases /100 000 livebirths. Assuming this case rate (i.e. 1 000 vertical HIV transmission cases /100 000 livebirths) was achieved with the 67.4% national viral suppression rate (during third trimester) that is reported in this study for the same year, a much higher viral suppression rate will be needed to reduce the current (1000 /100 000 live births) vertical HIV transmission cases rate to the targeted vertical HIV transmission case rate of 50/100 000 livebirths for elimination of vertical HIV transmission [32, 33].

As South Africa has a large population of reproductive age WLWH, one important strategy to eliminate pediatric HIV in South Africa is to ensure people living with HIV receiving ART (both men and women) have access to and awareness about safer conception methods and contraceptive services to prevent unintended pregnancy. With the rapid scale up of the ‘treat all’ policy, accessible and integrated contraceptive services targeting reproductive age women receiving ART could avert a significant number of unintended pregnancies among WLWH and the associated risk of vertical HIV transmission. In South Africa, the majority of pregnancies among WLWH are unintended and a substantial percentage of women receiving ART before pregnancy conceive on unsuppressed viral load, which shows missed opportunity for contraceptive and safer conception services at ART clinics [34, 35].

This study found unintended pregnancy was associated with lower probability of viral suppression among women who initiated ART before pregnancy. Other studies have found that unintended pregnancy may be associated with increased levels of stress and depression [36]. Lack of peer/social support during this stressful time, especially among women who do not have support systems and unmarried women, could result in poor treatment adherence and viral rebound [37]. Our data underscore the importance of engaging WLWH receiving treatment in reproductive health discussions so that their contraceptive needs are met and unintended pregnancies are prevented. It is also beneficial to provide peer support and counselling on stress coping mechanisms to women whose pregnancies are unintended as high stress levels associated with unintended pregnancy could lead to poor adherence to treatment and low viral suppression.

Despite the significant association between unintended pregnancy and unsuppressed viral load in this study, unintended pregnancy was not the only factor associated with viral suppression. In this study, we found that pregnant women who attended ANC in rural health facilities and those who had a pending syphilis test result at the third trimester (which was a proxy for late attendance of ANC) had higher odds of unsuppressed viral load compared to women attending ANC in urban health facilities and women who had a syphilis test result. This may indicate the need for a multifaceted approach to address multilevel factors influencing viral suppression. This includes health service level factors (such as human resources, and system challenges) that may create differences in quality of services between rural and urban facilities, and at individual level, knowledge and perception of pregnant women about early initiation of ANC. In addition, the literature shows addressing client level broader factors including management of co-morbidities, and addressing socioeconomic inequalities as well as strengthening service provision, such as early linkage and initiation of effective ART, regular viral load monitoring, and enhanced adherence counselling could improve viral suppression during pregnancy [38]. The transition to Dolutegravir (DTG)-based regimen could also improve viral suppression as it is highly effective and has a high genetic barrier to drug resistance.

This study has some limitations. Participants were enrolled from public health facilities, which may limit the generalizability of the findings to women attending private facilities. Given the cross-sectional design of the survey, causal association between unintended pregnancy and viral suppression could not be determined. The study has not collected information on the type of antiretroviral regimen participants received and the timing of ART initiation—although all participants initiated ART before pregnancy and were presumably on ART until the third trimester (i.e. for at least 7 months)—those who have been on ART for a longer duration are more likely to achieve viral suppression than those who started ART in the preceding months before pregnancy. This cross-sectional survey measured viral suppression at one point during pregnancy, which may not be representative of women’s continuous viral load burden. The definition for unintended pregnancy included the partner’s intention because the partner involvement (or lack of involvement) in the planning of pregnancy may influence the psychological and economic support the woman receives from the partner during pregnancy, and this may affect her adherence to ART and viral suppression. The pregnancy intention questions were self-reported by the woman—the woman’s understanding of her partner’s intention could be biased towards her own intention. In a separate analysis published recently from the same data, we showed the woman’s self-reported partner intention was largely similar to her intention [18]. Social desirability bias could also result in under reporting of unintended pregnancy. There may be risk factors /confounders that contribute to poor viral suppression that this study has not accounted adequately in the multivariable analyses: this may include co-infection with other STIs, disease stage, baseline viral load, the quality of adherence counselling and support provided during pregnancy, and psychosocial and socio-economic factors, which could contribute to poor viral suppression. Future studies could include these factors in assessing the association between viral suppression and unintended pregnancy.

In conclusion, overall, viral suppression among pregnant women in 2019 was below the UNAIDS target of 73% viral suppression. The UNAIDS target has been met among women who initiated ART before pregnancy. Low viral suppression was associated with unintended pregnancy among pregnant women who initiated ART before pregnancy. Routine assessment of fertility preferences and provision of contraceptive services at HIV clinics need to be strengthened to prevent unintended pregnancy as well as other factors that are associated with poor virologic suppression.

## Supporting information

S1 Table London measure of unplanned pregnancy (LMUP) questions and categorization of responses in the 2019 national antenatal HIV Sentinel Survey. (DOCX)
