# Supplementary material for: Association between viral suppression during the third trimester of pregnancy and unintended pregnancy among women on antiretroviral therapy: Results from the 2019 antenatal HIV Sentinel Survey, South Africa
Source: PLoS One. 2022 Mar 17;17(3):e0265124. doi: 10.1371/journal.pone.0265124 (PMC8929576; doi:10.1371/journal.pone.0265124)
Supplement: S1 Table — (DOCX) [file pone.0265124.s001.docx]

**S1 Table****. London measure of unplanned pregnancy (LMUP) questions and categorization of responses in the 2019 national antenatal HIV sentinel survey.**

| **Before I became pregnant** | **Just before I (mother) became pregnant** | | |
| --- | --- | --- | --- |
|  | I intended to become pregnant | My intention kept changing | I did not intend to become pregnant |
| The father of the child and I (mother) had agreed that we would like me to be pregnant | Both responses indicate intended (35.9%) | One response indicated intention undecided (1.1%) | One response indicated not intended (3.7%) |
| The father of the child and I (mother) had discussed having children together but hadn’t agreed for me to be pregnant | One response indicated intended (2.7%) | One response indicated intention undecided (1.8%) | Both responses not intended (16.9%) |
| We never discussed having child(ren) together | One response indicated intended (2.0% ) | One response indicated intention undecided (1.2%) | Both responses indicated not intended (34.7%) |

*The percentages in bracket shows the weighted distribution of participants’ response to the two LMUP questions. Grey dark: intended; Grey light: Ambivalent; White: unintended*
